# Typifications of some species names in Anthurium section Pachyneurium (Araceae)

**DOI:** 10.3897/phytokeys.178.65087

**Published:** 2021-05-27

**Authors:** Mel de Castro Camelo, Lívia Godinho Temponi, Simon Joseph Mayo, Marcus Alberto Nadruz Coelho, José Fernando A. Baumgratz

**Affiliations:** 1 Escola Nacional de Botânica Tropical, ENBT-JBRJ, Rio de Janeiro, RJ, Brazil Escola Nacional de Botânica Tropical Rio de Janeiro Brazil; 2 Universidade Estadual do Oeste do Panará, Herbário UNOP, Cascavel, PR, Brazil Universidade Estadual do Oeste do Panará Cascavel Brazil; 3 Department of Identification and Naming, Herbarium, Royal Botanic Gardens, Kew, Richmond, Surrey TW9 3AB, UK Department of Identification and Naming, Herbarium, Royal Botanic Gardens Richmond United Kingdom; 4 Instituto de Pesquisas do Jardim Botânico do Rio de Janeiro, JBRJ, Diretoria de Pesquisas, Rio de Janeiro, RJ, Brazil Instituto de Pesquisas do Jardim Botânico do Rio de Janeiro Rio de Janeiro Brazil

**Keywords:** *
Anthurium
*, Araceae, epitype, lectotype, Pothoideae, section *Pachyneurium*

## Abstract

During a taxonomic study of Anthurium sect. Pachyneurium, it was found that the names of four species required typification. Verification of the protologues and cited collections is discussed and typifications are proposed as follows: the illustration *Schott Icones Aroideae No. 465* is designated as the neotype of *A. affine* Schott. A lectotype is designated for *A. bonplandii* G.S.Bunting since the holotype, cited in the protologue at MY, was not found there. An epitype is selected for *A. solitarium* Schott because the lectotype illustration of J.M.C. Vellozo (*Flora Fluminensis t. 123*) lacks sufficient detail to determine it unambiguously to species in A. sect. Pachyneurium. A lectotype is selected for *A. glaziovii* Hook.f., a synonym of *A. solitarium*.

## Introduction

*Anthurium* Schott (Schott 1829) is a neotropical genus, the largest and possibly the most complex of the *Araceae* family, belonging to the subfamily Pothoideae (Cusimano et al. 2011), with 950 accepted species and many more which remain to be described (Boyce and Croat 2021 onwards; Cole et al. 2020). It is particularly diverse in perhumid cloud forests of Central and South America (Croat 1992; Mora et al. 2006).

In Brazil, the genus is represented by 152 species, of which 122 are endemic (Coelho et al. 2020). Classical field studies of the 19^th^ century in the country were carried out by many botanists, such as H.W. Schott, F. Sellow, L. Riedel, A. F. Regnell, A.F.M. Glaziou and C.A.M. Lindman, who made extensive living and dried collections later deposited in European herbaria and botanical gardens, from which many *Anthurium* taxa were described and named (Coelho and Mayo 2007).

Schott (1860), the pioneer in taxonomic studies of *Anthurium*, presented a classification of 28 infrageneric taxa named “*grex*” (plural “*greges*”). Many of these were later classified as sections and subsections by Engler (1878, 1898), who consolidated his revision in his final treatment, published in *Das Pflanzenreich* (Engler 1905), the last monograph of the genus at species level which was considered complete in its time.

Currently, the genus is subdivided into 20 sections (Carlsen and Croat 2019; Croat and Carlsen 2020). Only five of these have representatives in Brazil and the sections *Urospadix* Engl. and *Pachyneurium* (Schott) Engl. are those that circumscribe the majority of endemic species (e.g. Croat 1991; Coelho and Croat 2005; Gonçalves 2005; Temponi 2006; Coelho et al. 2009; Camelo et al. 2018a, b; Coelho and Valadares 2019; Hammes et al. 2020; Camelo et al. 2020; Coelho et al. 2020).

The first description of a species of section Pachyneurium was / published by Linnaeus (1763: 75) as *Pothos crenata* L. and later transferred to *Anthurium* by Kunth (1841: 75). Schott (1860) recognised 20 species in his grex *Pachyneurium* and Engler (1905) included 56 species in his A. sect. Pachyneurium. In the most recent revision, Croat (1991) included 114 species and 126 taxa and divided the section into two series, *Multinervia* Croat and *Pachyneurium* (Schott) Croat (Carlsen and Croat 2013). Subsequently, Carlsen and Croat (2019), in their recent study of sectional circumscriptions in *Anthurium*, proposed that series Multinervia should be recognised as a section because it was shown to be a monophyletic clade, all other species belonging to section Pachyneurium.

Anthurium sect. Pachyneurium is distributed throughout the Neotropics from Mexico to Argentina and distinguished by the dark brown colour of the leaves in dried specimens and the usually wide spacing of the rib-like secondary veins (more than 3 cm apart), which are more conspicuous than the inter-secondary veins (Croat 1991). The leaf blade has involute venation and eucamptodromous secondary venation (Croat and Sheffer 1983, 2002; Croat 1991). Probable synapomorphies of the section are the stem with very short internodes, the entire leaf blade and one ovule per locule (Temponi 2006).

Croat (1991) described three new species for Brazil from the Amazon Region in his taxonomic revision of A. section Pachyneurium. Taking into consideration taxonomic modifications and subsequent descriptions of new species, there are currently 23 taxa native to Brazil, occurring mainly in the Amazon and Atlantic Forest biomes (e.g. Croat 1991; Coelho and Croat 2005; Gonçalves 2005; Coelho et al. 2020). These species exhibit a wide range of morphological variability, partly due to a lack of detailed field observations and, consequently, incomplete published descriptions (Temponi 2005). However, the taxonomic difficulties of this group are also linked to the history of species diversification and delimitation in the genus (Carlsen and Croat 2013, 2019) and the generally very plastic vegetative characters used in morphological species delimitations (Mayo et al. 1997). These issues can lead to nomenclatural and taxonomic confusion and a lack of clarity in species limits.

Currently, Camelo and collaborators (in preparation) are carrying out revisionary studies of Anthurium sect. Pachyneurium in Brazil. During these investigations, some names have been found to lack type specimens, requiring typification to stabilise the nomenclature of their respective species. Therefore, we propose here one neotypification, two lectotypifications and one epitype.

## Materials and methods

In order to discover the types of H.W. Schott’s Brazilian species of *Anthurium*, it is, above all, necessary to make a detailed consultation of his published works, particularly his monograph *Prodromus Systematis Aroidearum* (Schott 1860). We also studied his magnificent collection of drawings, available in microfiche (Schott 1984) with the accompanying index (Nicolson 1984), the originals of which are deposited in the Archive Department of the Natural History Museum of Vienna, as well as black-and-white photographs made by the New York Botanical Garden. The coloured drawings represent plants cultivated by Schott himself in the Imperial Gardens of Schönbrunn Palace, Vienna, whereas the black and white drawings depict specimens from many European herbaria. Other publications and sources relevant to researching Schott types are Fenzl (1865), Wawra (1879), Riedl (1965a, b), Riedl and Riedl-Dorn (1988), Riedl-Dorn (1992), Grayum (1996), Coelho and Mayo (2007) and Mayo (2020a, b). We also studied the original black and white drawings of the “*Pothos*” (= *Anthurium*) species illustrated in the *Florae fluminensis* (Vellozo 1831), which are deposited in the National Library of Brazil in Rio de Janeiro.

Protologues of the treated species were studied using the databases of the Biodiversity Heritage Library (2020) (http://www.biodiversitylibrary.org/) and publications in scientific journals. Images of the type specimens were accessed through the database JSTOR Global Plants (2020) (http://plants.jstor.org/), GBIF.org (2020) (Global Biodiversity Information Facility; https://www.gbif.org/), REFLORA (2020) (http://reflora.jbrj.gov.br/) and the virtual herbaria of K, MO and NY (acronyms following Thiers 2020 continuously updated).

Typifications followed the nomenclature standards of the International Code of Nomenclature (ICN) criteria (Turland et al. 2018). The primary taxonomic literature sources consulted were the monographs of Schott (1860), Engler (1898, 1905) and Croat (1991). Complementary information on external morphology and phenology was made through observations of populations in the field and analysis of herbarium collections.

## Typification

### *Anthurium affine* Schott, Oesterr. Bot. Wochenbl. 5: 82. 1855

**Type.** (neotype, designated here), [icon] the original illustration H.W. Schott’s Icones Aroideae et Reliquiae No. 465, deposited in the Archive Department of the Natural History Museum of Vienna and available in the microfiche edition (Nicolson 1984; Schott 1984).

**Note.** The name *A. affine* was first cited as a *nomen nudum* by Schott (1829) in a short work in which he separated *Anthurium* from the broader Linnaean genus *Pothos* L. and mentioned this species as one of the plants flowering in the glasshouses of the Schönbrunn Gardens: “*Anthurium affine*. (*Schott.*) *Verwandter Blüthenschweif. Aus Brasilien. Aroideae*”.

The protologue given by Schott (1855: 82) to *A. affine* consists only of a passing remark during a discussion of the taxonomic problems then surrounding *A. hookeri* Kunth, written in German in an article entitled “Vermischtes”, which discusses various nomenclatural confusions in *Anthurium*. Schott observes that the name *A. hookeri* has been used for various cultivated plants, some with an elongated, long-pedunculate spadix and a basally cuneate leaf and others with basal lobes that are rounded or cordate at the petiole junction. However, *A. hookeri* is strikingly recognisable by its short spadix and short peduncle and is somewhat similar only to a Brazilian species, Schott’s *A. affine*, which, while certainly having a very short spadix, develops a long peduncle and is generally closer to *A. crassinervium* [“…*Anthurien mit langgestrecktem und langgestieltem Blüthenkolben und an der Basis keilig verlaufendem Blatte*, *andere mit unten rund oder herzförmig in den Blattstiel vereinigten Blattausbreitungen*, *sollen die echte*, *durch kurzstieligen*, *kurzen Blüthenstand so auffallend kenntliche Species kund geben*, ***die nur einigermassen mit einer brasilischen Art, unserem Anthurium affine, Aehnlichkeit hat (das zwar sehr kurzen Spadix, aber langen pedunculus entwickelt, überhaupt dem A. crassinervium näher steht***)…”].

The validating phrase of the *A. affine* name (marked in bold italics above), although short and in German, contrasts the inflorescence characters which distinguish it from *A. hookeri* and indicates a similarity to *A. crassinervium*. According to ICN (Art. 38.2), this descriptive phrase is sufficient to validate a name and therefore can be accepted as a valid original description. Later, Schott published a full description of *A. affine* in his monograph *Prodromus Systematis Aroideanum* (Schott 1860: 473–474). This description was clearly made from a living plant, since there are such details as leaf, spadix and berry colour and petiole cross-sectional shape, which are lost in dried specimens. In addition, Schott cites the locality and specimens he saw as follows “– Brasilia (S.) – *v.v.spont. et cult.*”, [“vidi vivam spontaneam et cultam”], an abbreviated Latin phrase meaning “I have seen living plants both wild and cultivated” (Stearn 1983: 292). Here, Schott makes it explicit that he had seen living plants in the field and associates his name (“S.”) with the locality “Brasilia”.

**Figure 1.**
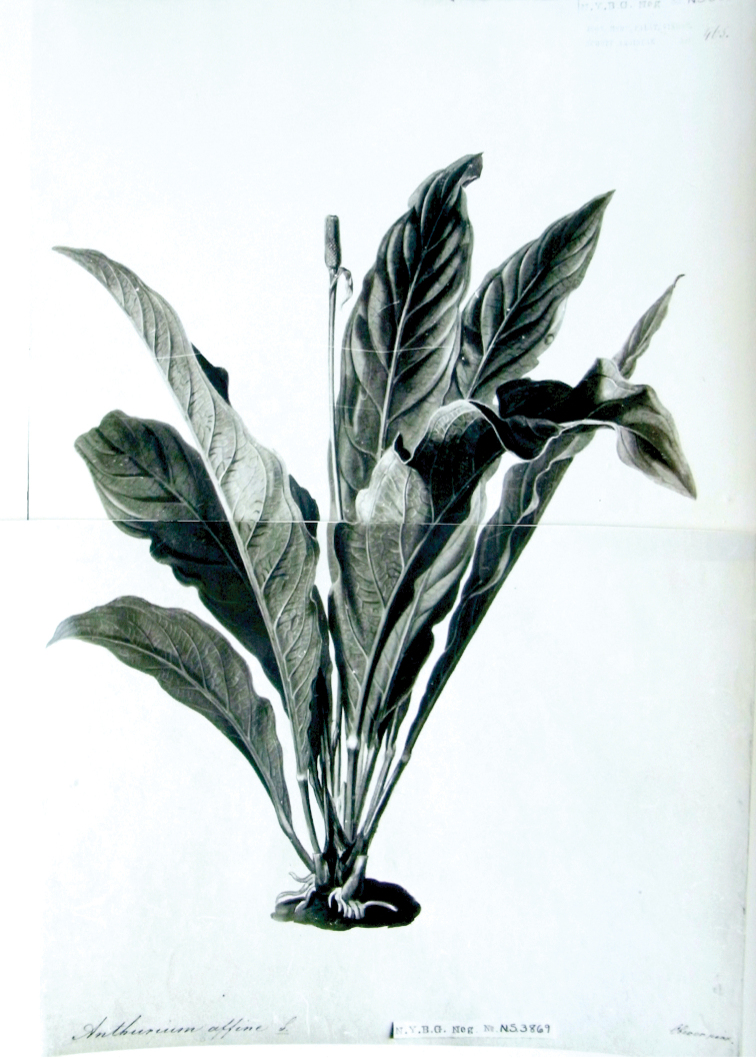
Neotype of *Anthurium affine*, the reproduction of the original coloured gouache *Schott Icones Aroideae n^o^ 465*, deposited in herbarium NYBG (NYBG Neg. N53869).

Croat (1991) in his revision of A. sect. Pachyneurium designated the drawing *Schott Icones Aroideae* no. 465 as the lectotype of *A. affine*, but without explaining the type designation. This is one of the five drawings of the species (nos. 463–467) preserved in Schott’s *Icones Aroideae et reliquiae* archive of illustrations (Nicolson 1984). They are coloured gouache *icones*, drawn and painted by the artists Oberer and Liepoldt (Riedl-Dorn 1992: 92) and no. 465 shows a single flowering plant as a whole, i.e. the complete habit of the plant with leaves and an inflorescence (Fig. 1). However, this lectotypification by Croat (1991) is vulnerable since Grayum (1996) and Coelho and Mayo (2007) argued that lectotypes should be chosen from materials known to have been in the author’s hands at the time of description. The drawing cited by Croat bears no date and so it cannot be established with certainty that Schott had it in his hands in 1855 at the time of the original valid description of *A. affine.* Consequently, this drawing must be considered a neotype rather than a lectotype, according to ICN (Art. 9.3 and 9.4).

Coelho and Mayo (2007) highlight that, in the case of binomials published by Schott himself, the coloured drawings (i.e. those depicting plants he cultivated in Vienna) are regarded as a source of neotypes rather than lectotypes, because they normally bear no date, making it impossible to determine whether they had been drawn before or after the publication of the names. They also point out that, in the absence of a type specimen, Schott’s drawings may be the only remaining evidence of the type. There is no evidence that the drawings of *A. affine* were available to Schott in 1855 when this species was validly published. Consequently, Icon no. 465 cannot be considered as the lectotype. Since no type specimen has been found, we designate here the illustration *Icones Aroideae et Reliquiae* No. 465 as the neotype of *A. affine* because it agrees well with the description of the spadix and peduncle length presented in the protologue and later complete description (Schott 1855, 1860).

### *Anthurium bonplandii* G.S.Bunting, Acta Bot. Venez. 10: 267 (1975)

**Type.** Venezuela. [Amazonas], Dept. Atures, Rio Orinoco near Siquita, between Isla Castillito and San Fernando de Atabapo, 1969, 100–140 m alt., *G.S. Bunting*, *L. Akkermans et J. van Rooden 3676* (holotype, MY!: not located); (lectotype, designated here: K barcode K000434112!; (isolectotypes: K barcode K000434111!, NY barcodes NY00133732!, NY00133733!, NY00133734!, NY000133735!).

**Note.***Anthurium bonplandii* was described by Bunting (1975) as a new species, based on a single collection, in honour of Aimé Bonpland, who travelled with Alexander von Humboldt in regions of Venezuela where this species occurs. The author cited the specimen *G.S. Bunting*, *L. Akkermans et J. van Rooden #3676* (MY) as the holotype, without mentioning any isotype. According to the curator of Maracay Herbarium (MY), all type material of *A. bonplandii* was sent to K and NY sometime after the species’ publication. No type material was found at MY herbarium, but there are four sheets of this collection at NY and two at K. These specimens are all marked on their original labels as “Typus”, but none with the designation holotype. In 1987, the NY sheets were labelled by J. Burgess as isotypes, probably following Bunting’s “holotype” designation for MY in the original description.

**Figure 2.**
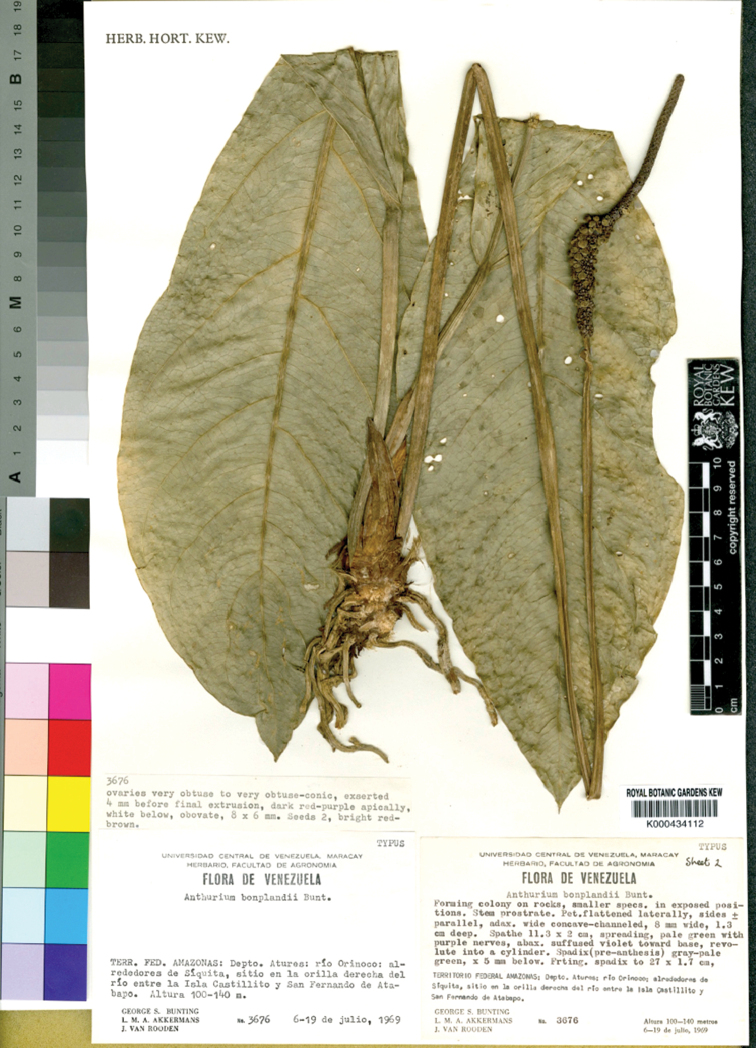
Lectotype of Anthurium bonplandii subsp. bonplandii in K herbarium (K000434112).

We cannot be sure if one of them is the original holotype designated by Bunting, but it is clear from their original labels that they are isotypes, according to ICN (Art. 9.5). Since the holotype has not been found, we have designated *G.S. Bunting*, *L. Akkermans et J. van Rooden #3676* (K000434112) (Fig. 2) as the lectotype, chosen from amongst the isotypes, in accordance with ICN (Art. 9.3). This specimen was selected because it is the single most complete preserved specimen, including the stem with connected vegetative and reproductive structures and agrees with the protologue description.

### *Anthurium solitarium* Schott, Prodr. Syst. Aroid. p. 478. 1860.

≡ *Pothos solitarius* Vell., Fl. flum. text. p. 390, tab. 123 (“122”). 1881, as “*solitaria*”, *nom. illeg*., [*Pothos solitarius* (“*solitaria*”) Vell., Fl. flum. t. 123 (“122”). 1831 [“1827”], *nom. non valid. publ.*].

= *Anthurium glaziovii* Hook. f., Bot. Mag. t. 6833. 1885. Type: BRAZIL. Rio de Janeiro, *Glaziou 188*. (lectotype, designated here: 3 sheets, part 1 K barcode K000434142!, part 2 K barcode K000434143!, part 3 K [barcode K000434144]!).

= *Anthurium nobile* Engl., Bot. Jahrb. Syst. 25: 366. 1898. Type: BRAZIL. Rio de Janeiro, *Glaziou 9039*. (holotype: B barcode B100242945!; isotype: P barcode P00748724!).

**Type.** Brazil. Rio de Janeiro, Santa Cruz (“*Regii Praedii S. Crucis*”), illustration of “*Pothos solitaria*” in J.M.C. Vellozo, *Flora Fluminensis*, t. 123 (1831 [“1827”]) (lectotype, designated here): original parchment illustration of *Flora fluminensis* t. 123 in the Manuscript Section, Biblioteca Nacional, Rio de Janeiro, indexed under digital object code mss1198658_127!) (epitype, designated here: BRAZIL. Rio de Janeiro, Nova Friburgo, PE dos Três Picos, picada para os Três Picos, 22°20'9"S, 42°43'0"W, Floresta Ombrófila Densa Altomontana, 05 April 2016, *M. Nadruz et al. 3064* (RB barcode RB01111541!).

**Note.** The plates of Araceae were published in volume 9 of the *Flora Fluminensis* of José Mariano da Conceição Veloso (Vellozo 1831), long before the text of the species descriptions (Vellozo 1881). Plate 123 shows a species of Anthurium sect. Pachyneurium and bears the name “*Pothos solitaria*”, but this was a *nomen nudum* at this date, according to ICN (Art. 38, 38.8 and 38.9), since the plate lacks both an accompanying description and any analytical figure.

Schott (1860) then validly published the name *Anthurium solitarium*, citing only “*Brasilia*. (*Flor. Flum.*)”. This is an indirect, but unambiguous reference to Vellozo’s plate 123 of “*P. solitaria*” in the *Flora Fluminensis*, since the original 1831 volume 9 of the plates was the only published form of the Araceae then available. Schott’s short description seems clearly to be based on the leaves and inflorescence shown in plate 123, which can thus be regarded as the type of Schott’s binomial name. According to ICN (Art. 6.9 and 6.10), the name *Anthurium solitarium* therefore cannot be regarded as a new combination, with *Pothos solitarius* (as “*Pothos solitaria*”) as its basionym, but is rather to be treated as the name of a newly-published species.

The name *Pothos solitarius* (as “*Pothos solitaria*”) was eventually validly published in Vellozo (1881: 390), with a description of the plant’s leaves, inflorescence, locality, habitat and rupicolous habit (“*Habitat fruticetis Regii Proedii S. Crucis supra lapides*”), besides the original illustration. However, *P. solitarius* is an illegitimate name, being superfluous according to ICN (Art. 52.1 and 52.2), due to its later publication and inclusion of the eligible type of *A. solitarium*Schott (1860), the original plate of “*P. solitaria*” (Vellozo 1831: tab. 123).

The locality, cited by Vellozo, corresponds to one of those listed by Lima (1995) in his study of Vellozo’s Leguminosae. The site of the “*Regium Praedium Sanctae Crucis*” – literally “Royal Estate of Santa Cruz” – lies in the modern Santa Cruz District of Rio de Janeiro, where, during the colonial period, there was an estate owned by the Crown and used for the cultivation of *Camellia sinensis* (L.) Kuntze, popularly known as Indian tea (*chá-da-índia*). In addition, Vellozo cites “*Habitat fruticetis … supra lapides*”, meaning “Occurring in shrubby vegetation … on rocks”. In the Municipalities (Mangaratiba, Angra dos Reis and Paraty) adjoining Santa Cruz Municipality, *A. solitarium* is very common as a rupicolous plant growing on rocks by the sea.

**Figure 3.**
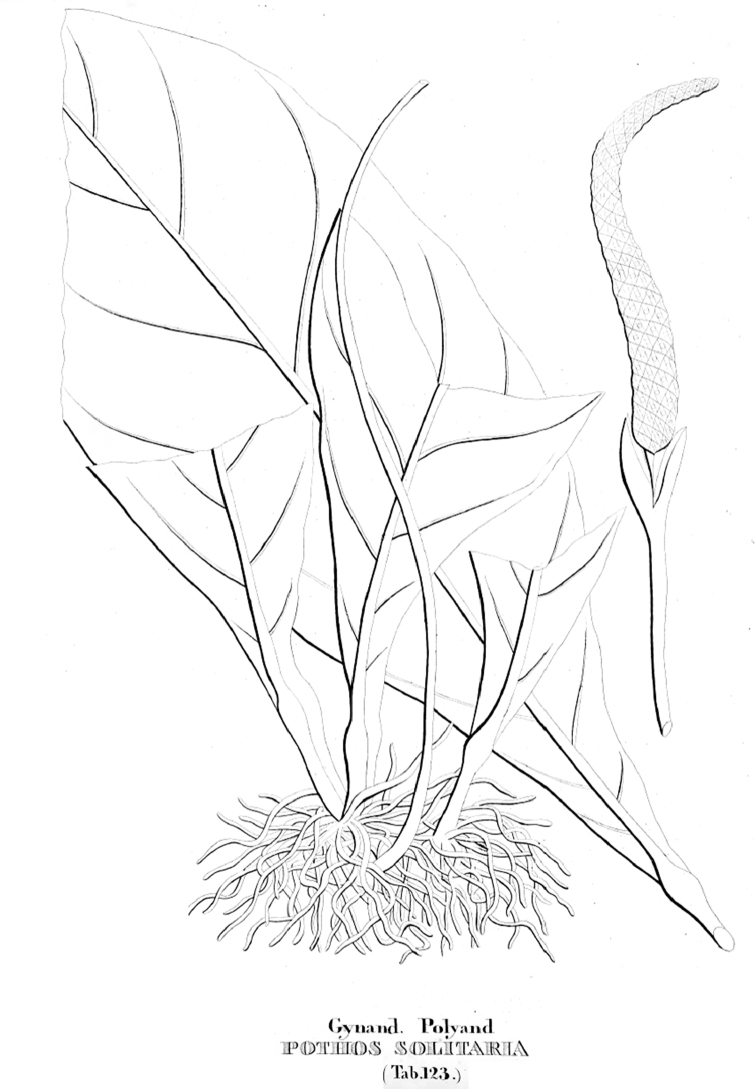
Lectotype of *Anthurium solitarium* (Garamond *Pothos solitarius*), the illustration “Pothos solitaria” in Vell., Fl. Flum. t. 123. 1831.

Vellozo’s plate 123 (Fig. 3) shows only very few characteristics in common with *A. solitarium*, such as the presence of numerous roots and the slightly wavy leaf blade margins. However, the most diagnostic characteristic of this species – the pendent inflorescence – is not shown; instead the inflorescence is shown on the contrary as an erect structure. From the modern taxonomic standpoint, the plate is ambiguous and inconsistent, because it shows the leaves cut in half, does not include the complete shape of the leaf blade and details of the spathe are lacking. According to current understanding of *Anthurium solitarium*, the spathe and spadix are almost the same size and the inflorescence is pendent, both characteristics being important for the circumscription of the species. Consequently, this illustration could be confused with certain other species of A. sect. Pachyneurium from southeastern Brazil, i.e. *A. leonii* E.G. Gonç. and *A. santaritense* Nadruz & Croat (Gonçalves 2005; Coelho and Croat 2005).

Engler (1898: 352) confused *A. solitarium* with *A. affine*. In the synonymy of the latter, he included the names *P. solitaria* and *A. solitarium* with question marks, indicating his doubt. This can also be seen in his incorrect determinations, as *A. affine*, of specimens of *A. solitarium* deposited in various herbaria. Two examples are: a drawing and specimen of: (A. Engler Araceae exsiccatae et illustratae no. 295 [K!]) from a plant cultivated in the Breslau (today Wroclaw) University botanic garden and the specimen *Glaziou #9040* (F0BN011838), collected in Rio de Janeiro and deposited at the Field Museum of Natural History.

Despite this earlier confusion, *A. solitarium* differs markedly from *A. affine* in its morphology, ecology and geographical distribution. Current revisionary studies of Anthurium sect. Pachyneurium (Camelo et al., in prep.), have revealed that four species occur in the Southeast Major Region of Brazil: *A. affine*, *A. leonii*, *A. santaritense* and *A. solitarium*, of which the taxon most closely related to *A. solitarium* is *A. leonii*. *Anthurium solitarium* differs 1) from *A. leonii* by the vinaceous to brownish, cylindrical to 1–ribbed peduncle, the spathe purple or brown on both surfaces and the whitish pollen grains (vs. peduncle greenish, cylindrical, never ribbed, spathe lilac on adaxial surface, greenish on abaxial surface and yellowish pollen grains) (Camelo et al., in prep.); 2) from *A. santaritense* by the obovate leaf wider than 21 cm and the pale purple or brown, sessile spadix > 11 cm long (vs. narrowly lanceolate leaf blade up to 20 cm wide, spadix < 10 cm long., lilac, with 0.3–2.5 cm stipe); and 3) from *A. affine* by an obovate leaf blade with non-wavy to slightly wavy margins (vs. elliptic leaf blade with strongly wavy margins), pendent inflorescence (vs. erect inflorescence), elongated purple or brown spathe (vs. spathe elliptic to ovate, green or green with vinaceous striations) and a slender, tapered, pale purple or brown flowering spadix (vs. cylindrical to clavate, yellowish-green to yellow flowering spadix) (Camelo et al., in prep.).

*Anthurium leonii* is a common rupicole in humid areas of the Atlantic Forest. *Anthurium santaritense* and *A. solitarium* are common epiphytes or rupicoles in humid areas, montane forest or in restingas of the Atlantic Forest Region in south-eastern Brazil. In contrast, *A. affine* is a rupicole in *campos rupestres* and *cerrado* or occurs as a terrestrial plant in *campos* in *restingas* of the Atlantic Forest Region (Coelho et al. 2020; Camelo et al., in prep.).

**Figure 4.**
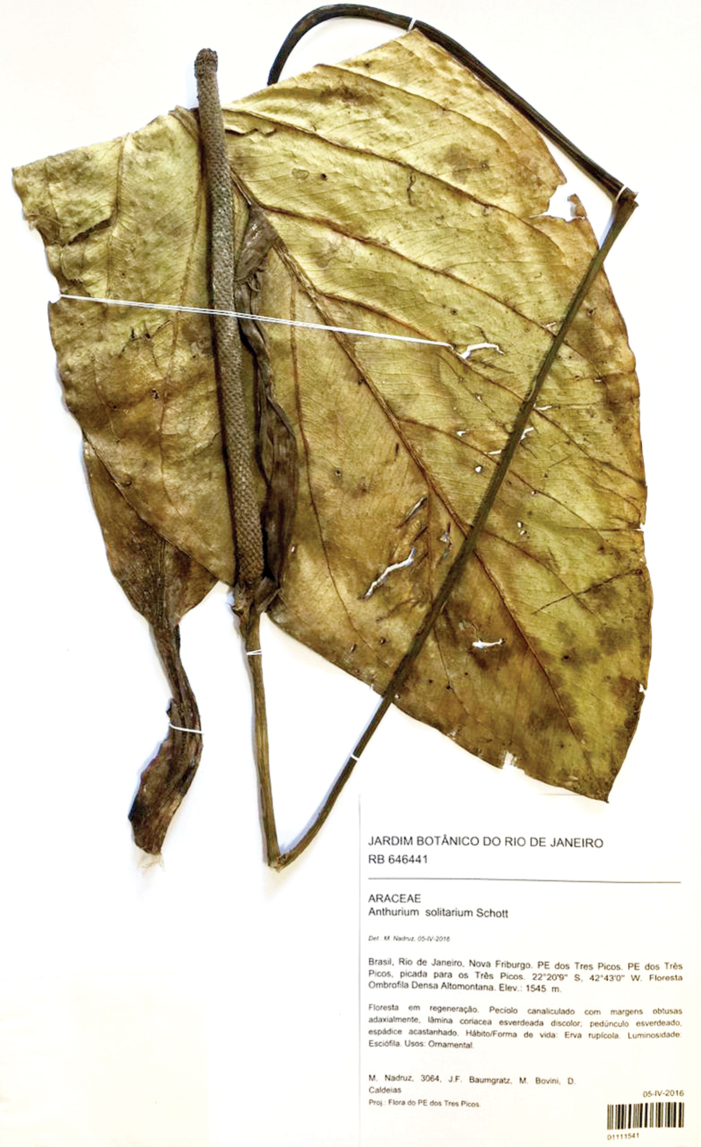
Epitype of *Anthurium solitarium* (≡ *Pothos solitarius*) in RB herbarium (RB01111541).

Following the recommendation of ICN (Art. 9.9) to correct the taxonomic ambiguity of Vellozo’s plate, we designated here as epitype of *A. solitarium* Schott, the specimen *M. Nadruz et al. 3064* (RB01111541) (Fig. 4). This specimen is complete and consistent with the original description. Since there is no material collected in the type location (the City of Rio de Janeiro), we have selected a specimen collected in the Atlantic Forest Region of Nova Friburgo Municipality, in Rio de Janeiro State.

The original description of *A. glaziovii* Hook.f. was based on a living plant sent by A.F.M. Glaziou in 1880, from Rio de Janeiro to the Royal Botanic Gardens, Kew, where it flowered in June 1881. Hooker (1885) noted in the plant’s description that its exact origin was unknown, but was presumably Rio de Janeiro. Three sheets of this cultivated plant are preserved at K, prepared by N.E. Brown and labelled in his handwriting as “Anthurium Glaziovii Hook. f.! Type specimen of Bot. Mag. t. 6833! Rio de Janeiro, Glaziou Hort. Kew June 8. 1881 labelled “Glaziou No. 3 188/1880” N.E. Brown”. Therefore, we designated this specimen as the lectotype, referring to it as *Glaziou #188* (K) and consisting of the following three elements: sheet 1: (K000434142), sheet 2: (K000434143) and sheet 3: (K000434144).

The holotype of *A. nobile* was located at B herbarium (B100242945) and an isotype at P herbarium (P00748724). Based on the study of these type materials, the application of the names *A. glaziovii* and *A. nobile* is confirmed as heterotypic synonyms of *A. solitarium*, as previously accepted by Croat (1991).
